# The effects of a dietitian‐supported multidisciplinary nutrition intervention on optimizing nutrition care in older patients with hip fracture and at nutrition risk—A quality improvement study

**DOI:** 10.1002/ncp.70049

**Published:** 2025-10-08

**Authors:** Tina Munk, Anne Marie Beck, Cecilie M. Møller, Frederikke E. Pudselykke, Guro Ø.H. Mikkelsen, Heidrun T. Filtenborg, Trine S. Pedersen, Jens Peter Alva‐Jørgensen, Anne W. Knudsen

**Affiliations:** ^1^ Dietetics and Nutrition Research Unit, EATEN Copenhagen University Hospital – Herlev and Gentofte Herlev Denmark; ^2^ Department of Orthopedic Surgery Copenhagen University Hospital – Herlev and Gentofte Herlev Denmark; ^3^ Department of Internal Medicine Copenhagen University Hospital – Herlev and Gentofte Herlev Denmark

**Keywords:** adult, life cycle, nutrition, nutrition assessment, nutrition support practice, nutrition support teams, outcomes research/quality, research and diseases, weight loss

## Abstract

**Introduction:**

A 1‐day cross‐sectional study at our hospital found that only 22% of patients with hip fractures at nutrition risk met their energy and protein requirements during hospitalization. This study aimed to test whether closer collaboration between a clinical dietitian and ward staff, guided by the Model for Improvement, could optimize nutrition care for hospitalized older patients with hip fractures at nutrition risk.

**Method:**

A dietitian was embedded to facilitate staff‐led enhancements in nutrition care at an orthopedic ward in from September to December 2024. Two Plan‐Do‐Study‐Act cycles were implemented. Cycle 1 focused on nutrition documentation. Cycle 2 targeted nutrition intake. The primary outcome was the proportion of patients meeting individual energy and protein requirements (≥80%). Secondary process indicators were (1) ≥80% of patients screened using Nutrition Risk Screening 2002, and (2) ≥80% of at‐risk patients with intake documented in the medical record. Preintervention data served as the baseline.

**Results:**

The primary outcome was achieved, with 80% (8 of 10) of patients meeting both energy and protein requirements, a significant improvement from 22% (2 of 9) at baseline (*P* < 0.05). Documentation of nutrition risk increased from 10% (1 of 10) to 80% (8 of 10) (*P* < 0.01), and intake documentation improved from 30% (3 of 10) to 100% (10 of 10) (*P* < 0.01).

**Conclusion:**

This quality improvement study demonstrates that applying the Model for Improvement to integrate a clinical dietitian into ward practice strengthened interdisciplinary nutrition care and led to measurable gains in screening, documentation, and nutrition intake among older patients with hip fractures at nutrition risk.

## BACKGROUND

Hip fractures among older adults represent a critical turning point in health trajectories, often resulting in loss of independence, prolonged hospital stays, and increased mortality risk.^1^, ^2^ Despite advancements in surgical and rehabilitation protocols, outcomes remain suboptimal for many patients, particularly those who are malnourished or at nutrition risk upon admission.^3^, ^4^ Malnutrition is highly prevalent in this patient population, with studies reporting rates ranging from 13% to 63%, depending on the assessment method.^5^ This nutrition vulnerability is not merely a comorbidity, but a modifiable risk factor with profound clinical implications. Malnourished patients with hip fractures are at significantly higher risk of complications, hospital readmission, prolonged recovery, and even 5‐year mortality.^2^, ^6^ In a recent prospective study, impaired nutrition status was found to be an independent prognostic factor for postoperative complications, discharge outcomes, hospital length of stay, and long‐term survival.^2^

In a cohort study conducted in September 2023 at a tertiary metropolitan hospital in Denmark, we found that only 22% of patients with hip fractures at nutrition risk (*n* = 9) met their estimated energy and protein requirements during hospitalization (unpublished data). The findings are supported by a former study from our hospital, which found that low protein and energy intake were common during hospitalization, with only a minority of patients meeting recommended intakes, even when oral nutrition supplements (ONS) were offered.^7^

Current literature highlights the potential of nutrition interventions in this context. Oral supplementation and structured dietary support can reduce complications, preserve functional capacity, and support recovery.^1^, ^3^, ^8^ This is recognized in recent guidelines, such as those from The European Society for Clinical Nutrition and Metabolism (ESPEN), which recommend that nutrition interventions must be individualized, comprehensive, and part of a multimodal and multidisciplinary team approach in older patients with hip fractures.^9^ Yet, translating these guidelines into practice remains a challenge.^10^ Inconsistent implementation, poor adherence to guidelines, and lack of interdisciplinary collaboration often limit their real‐world effectiveness.^1^, ^4^, ^5^ A global survey found that <20% of clinicians routinely provided high‐protein meals, ONS, and patient education to older patients with hip fractures, despite clear recommendations.^10^ Common barriers included inadequate staff training, unclear responsibilities, and poor integration of nutrition into clinical routines, challenges that were already identified in an early European report and continue to be highly relevant today.^11^

Nutrition care is widely recognized as a multidisciplinary responsibility, yet several authors emphasize that success depends on coordinated collaboration and clearly defined roles, with clinical dietitians playing a key role.^5^, ^12^, ^13^ However, involvement of clinical dietitians is often fragmented or underused. A study from our hospital demonstrated that when a clinical dietitian led an intervention combining individualized counseling with a protein‐enriched menu, 90% of hospitalized patients at nutrition risk met their nutrition targets, compared with 66% without counseling.^14^ Although the study did not focus on interdisciplinary collaboration, it illustrates that assigning dedicated clinical responsibility for nutrition can help close key implementation gaps. These findings point to the need for a more systematic approach, in which clinical dietitians not only contribute specialized expertise but also support and coordinate the interdisciplinary nutrition effort to ensure consistent, integrated care.

The Model for Improvement provides a structured approach to test changes and evaluate their impact and is widely used in healthcare quality improvement initiatives.^14^, ^15^ However, whether the model can be used to optimize nutrition care of patients with hip fractures has, according to our knowledge, not been examined before.

This led to the present study, in which the overall aim was to test whether closer collaboration between a clinical dietitian and ward staff, guided by the Model for Improvement, could optimize nutrition care for hospitalized older patients with hip fractures at nutrition risk.

## MATERIALS AND METHODS

### Study design and setting

This study was designed as a pragmatic action research project, with the Model for Improvement (Plan‐Do‐Study‐Act, PDSA) used as the guiding framework for the improvement process.^14^, ^15^ Action research was selected for its participatory and iterative nature, wellsuited to facilitating organizational change in clinical settings.^14^ The study was conducted at the Department of Orthopedic Surgery in collaboration with the Dietetic and Nutrition Research Unit, EATEN, at Herlev Hospital, from September 2024 to December 2024. During this period, a clinical dietitian was embedded to facilitate staff‐led enhancement of nutrition care at the orthopedic ward (30 h/week). This time allocation was provided from improvement resources within the hospital's dietetic and nutrition research unit and was in addition to the ward's usual staffing. Before the intervention, the orthopedic ward had no dedicated dietitian. As is common in Denmark, dietitians are only involved through referral, meaning they act as consultants when ward staff identify a need.

### Characteristics of participants and data sources

Demographic data, including sex, age, and body mass index (BMI), were retrieved from the medical journal. Eligible participants were patients admitted and surgically treated for hip fracture who were at nutrition risk, defined as Nutrition Risk Screening 2002 (NRS‐2002) score ≥3. Patients were excluded if they had declined participation in research or were in end‐of‐life care.

Data were collected at four time points: (1) a ward‐based cohort study in September 2023 (*n* = 9), which revealed that only 22% of patients achieved ≥75% of their estimated energy and protein requirements, and these results provided the main incentive for initiating the present quality improvement project and served as contextual baseline intake data; (2) an immediate preintervention baseline in September 2024 (*n* = 10), which included process indicators only; (3) after the first intervention period in October 2024 (*n* = 10), when process indicators were collected; and (4) after the second intervention period in December 2024 (*n* = 10), when the primary outcome (nutrition intake) was assessed. This design allowed comparison across a historical cohort baseline, an immediate preintervention baseline, and the two intervention phases. The embedded dietitian who facilitated the interventions also collected data. Although this dual role may introduce risk of bias, it was considered the most feasible approach in the context.

### Steps in the Model for Improvement

The Model for Improvement consists of two main components. Firstly, three guiding questions were used to define the aim, establish measures, and identify changes. Secondly, the iterative PDSA cycles were applied to test and refine the interventions.^15^

The primary aim was for at least 80% of patients at nutritional risk achieved ≥75% of their individual energy and protein requirements. Energy requirement was set to 27 kcal/kg body weight, and protein requirement was set to 1.2 g protein/kg body weight/day.^16^ Meeting requirements was pragmatically defined as achieving ≥75% of the estimated individual needs. Although the nutrition goal should ideally be to reach 100% of requirements, both ESPEN and the Danish Health Authority highlight that intake below 75% is insufficient, and previous studies in hospitalized older adults have therefore used ≥75% as a pragmatic and clinically meaningful threshold of adequacy.^7^, ^9^, ^16^ Dietary intake was assessed once per patient by the embedded dietitian using a 24‐h recall on day 4 after surgery. This time point was chosen to ensure comparability with the 2023 cohort study, which used the same method and timing. Data were entered into an Excel spreadsheet developed for the project, which calculated energy and protein intake and expressed these as a percentages of individual requirements. The 80% target was informed by a previous in‐house study, in which 90% of hospitalized patients at nutritional risk met their nutritional requirements when supported by intensive dietitian‐led counseling in combination with a high‐energy, high‐protein food concept.^17^ However, the target was set lower in the present study because of a different methodological approach, focusing on improving interdisciplinary collaboration and ward‐level nutrition workflows, rather than delivering intensive, dietitian‐led counseling alone.

To support the primary aim, two secondary process indicators focusing on documentation were defined: (1) ≥80% of patients should be screened for nutrition risk using the NRS‐2002 tool within 48 h of admission, and (2) ≥80% of those identified as nutritionally at risk should have their intake documented in the medical record. The process indicators (nutrition risk screening and intake documentation) were assessed by reviewing the electronic medical record. Each patient was evaluated once to determine whether screening and documentation had been implemented after intervention 1. The process indicator “documentation of intake” was derived from routine staff recording of nutrition intake in the medical record, not on the 24‐h recall.

**Figure 1:**
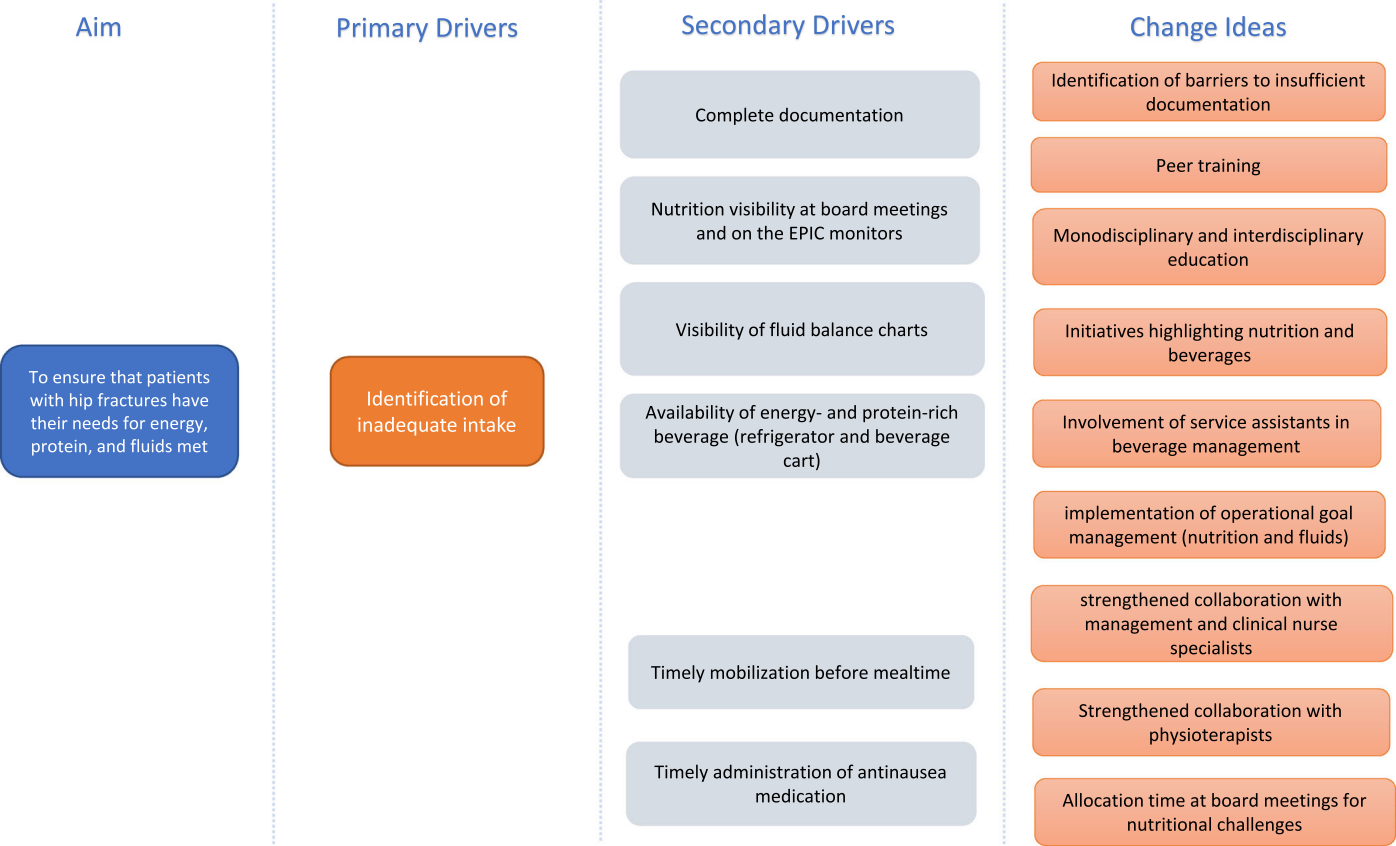
Driver diagram illustrating the aim, drivers, and change ideas codeveloped during a participatory staff workshop. EPIC, Electronic Patient Information Center.

In line with the third guiding question of the Model for Improvement (“What changes can we make that will result in improvement?”), barriers and potential solutions were identified through a structured process. Initially, the embedded dietitian conducted a 2‐week observation period and engaged in informal conversations with staff. This was followed by staff interviews, meetings with the clinical nurse specialist, departmental managers, and the research team, and, finally, a participatory workshop. The workshop was led by the embedded dietitian in collaboration with the clinical nurse specialist and included ward nurses, social and healthcare assistants, ward nurse managers, and service staff. Its purpose was to validate and prioritize barriers and to co‐develop strategies considered feasible for implementation. Improvement was defined as a statistically significant increase in nutrition intake or in the selected process indicators compared with baseline values. Based on the prioritized strategies, a driver diagram (Figure 1) was developed to guide the intervention design.

### The PDSA cycles

Based on the prioritized strategies and the identified change areas in the driver diagram, we structured the interventions around two PDSA cycles. These cycles targeted key areas considered feasible to address within the limited implementation period (mid‐September to mid‐December 2024). The aim was to test and refine two distinct interventions designed to optimize nutrition care in practice.

### PDSA cycle 1—Strengthening nutrition documentation

The first PDSA cycle focused on the two defined process indicators, improving nutrition risk screening and dietary intake registration, because these were considered critical for achieving the primary outcome. The interventions included dietitian‐led education sessions and peer training for nurses and physicians. The training addressed how to conduct nutrition risk screening, resolve challenges related to using the electronic medical record system, and emphasized the clinical importance of nutrition. It also aimed to establish a shared and meaningful nutrition language, such as using terms such as “achieved percentage of requirement,” rather than vague phrases like “ate well.” Further, the embedded dietitian provided individualized nutrition guidance to at‐risk patients and supported efforts to improve visibility and interdisciplinary communication of nutrition status. This included displaying nutrition intake data on the electronic patient dashboard for use during board rounds discussions and adding intake indicators such as achieved percentage of requirement to patient information boards to prompt nutrition‐related conversations at clinical ward rounds.

### PDSA cycle 2—Improvement in nutrition intake

In the second PDSA cycle, we focused on the primary aim of achieving adequate energy and protein intake, particularly by promoting the use of nutrient‐dense beverages because this had been identified as an important area for improvement on the ward.^7^ This included encouraging the use of milk‐based drinks instead of juice‐based options and emphasizing the appropriate use of ONS for patients with low appetite. Beverages can contribute not only to energy and protein intake but also to overall fluid balance. The intervention involved training of catering assistants in the ward by the dietitian. The training emphasized the importance of nutrition in the recovery of older patients with hip fractures and focused specifically on how beverage choices can influence nutrition adequacy. For example, the embedded dietitian highlighted how selecting milk‐based drinks instead of juice‐based options could significantly increase protein intake. In parallel, nursing staff were trained to pay greater attention to beverage‐related nutrition care and to ensure the ready availability of appropriate drink options on the ward. To make the training more engaging, a themed “Happy Hour” session was held, featuring tastings of milk‐based beverages, including medical ONS, as well as a nutrition quiz to reinforce key messages in a fun and interactive way. During the intervention period, the dietitian offered tailored nutrition counseling to patients and their relatives while also serving as a resource for staff through ongoing consultation and interdisciplinary support.

### Study and act

**Figure 2:**
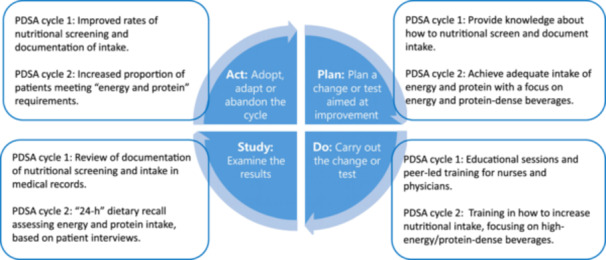
Illustration of the phases of the PDSA cycle, including the two distinct interventions designed to optimize nutrition care in clinical practice. PDSA, Plan‐Do‐Study‐Act.

Following each PDSA cycle, results were analyzed to determine whether the interventions had led to improvements in nutrition care. Reflections on implementation challenges and lessons learned informed necessary adjustments. Interventions found to be effective were sustained for the remainder of the study period. An overview of the phases is presented in Figure 2.

### Statistics

Descriptive statistics were used, and data are presented as median (interquartile range) and as *n* (%) for frequencies. Categorical variables were analyzed using Fisher's exact test, suitable for small samples. To compare differences between baseline cohort data, baseline preintervention data, and data collected after each intervention, a Kruskal‐Wallis test was used. Differences between baseline cohort data and after the second intervention were analyzed using a Mann‐Whitney *U* test. A *P* value < 0.05 was considered statistically significant. The statistical analyses were performed using SAS Enterprise Guide version 7.1 (SAS Institute, Cary, NC).

### Ethical approval

The Hospital Board of Directors approved the study as a quality improvement project (ID: 4047115), hence no informed consent to participate from the patients was needed. However, all electronic medical records were reviewed to determine if any patients had declined to participate in this type of study.

## RESULTS

### Patient characteristics

Patient characteristics gathered at the different time points (two different baseline periods and after the two interventions) are summarized in Table 1. Median age and BMI were comparable across groups, although some variation in sex distribution was observed.

**Table 1:** Patient characteristics.

Time point	Baseline: cohort data (September 2023; *n* = 9)	Baseline: preintervention (September 2024; *n* = 10)	After 1 intervention (October 2024; *n* = 10)	After 2 interventions (December 2024; *n* = 10)
Female sex, *n* (%)	5 (56)	3 (30)	1 (10)	4 (40)
Age, median (IQR), years	82 (81–86)	84 (78–92)	83 (75–88)	84 (82–87)
BMI, median (IQR), kg/m^2^	24 (22–25)	23 (20–25)	25 (22–26)	21 (20–23)

*Note*: Baseline cohort data (September 2023) was collected as part of a ward‐based study conducted before the planning of the present project. Preintervention baseline data (September 2024) were collected immediately before initiating the interventions. No difference between groups was found. Differences between groups are analyzed by a Fisher's exact test or a Kruskal‐Wallis test as appropriate.

Abbreviations: BMI, body mass index; IQR, interquartile range.

### Secondary process indicators

After the first PDSA cycle, 80% (8 of 10) of the patients were screened by NRS‐2002. This was a significant improvement compared with baseline (10% to 80%; *P* < 0.01). In addition, 100% (10 of 10) of the at‐risk patients had their intake documented in the medical records, which was also a significant improvement (30% to 100%; *P* < 0.01) compared with preintervention baseline data.

### Primary outcome—Nutrition intake

**Figure 3:**
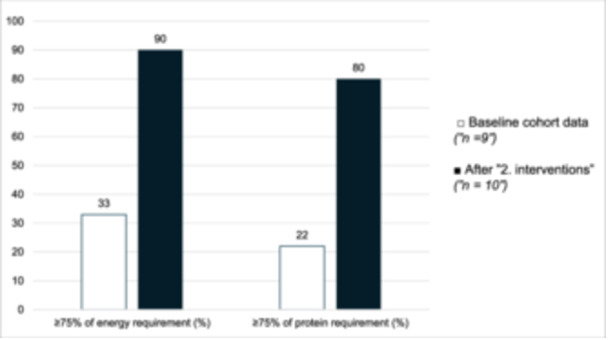
Proportion of patients meeting ≥75% of energy and protein requirements at baseline (*n* = 9) and after the "2. interventions‐" (*n* = 10). *P* < 0.05 for both.

After the second PDSA cycle, 80% (8 of 10) of patients met both their estimated energy and protein requirements, representing a significant improvement compared with the cohort baseline data, in which only 22% (2 of 9) achieved this target (*P* < 0.05) (Figure 3). Further details on nutrition intake are provided in Table 2 and Figure 3.

**Table 2:** Nutrition intake.

Time point	Baseline cohort data (September 2023; *n* = 9)	After 2 interventions (December 2024; *n* = 10)
Energy requirement, median (IQR), kcal/d	1782 (1404–1971)	1619 (1350–1727)
Protein requirement, median (IQR), g/d	86 (68–95)	71 (60–80)
Energy intake, median (IQR), kcal/d	946 (812–1560)	1445 (1243–1693)
Protein intake, g/d, median (IQR), g/d	38 (26–47)	56 (54–60)*
Energy requirement, median (IQR), %	63 (46–98)	92 (83–105)
Protein requirement, median (IQR), %	42 (38–58)	85 (75–93)**

*Note*: Interquartile range energy and protein intake was estimated using 24‐h recall of dietary intake. Energy requirement: 27 kcal/kg body weight. Protein requirement: 1.2 g/kg body weight. Baseline cohort data from a study conducted in September 2023 was used as the control. Comparisons between baseline and postintervention values were performed using the Mann‐Whitney *U* test for continuous variables and Fisher's exact test for categorical variables.

Abbreviation: IQR, interquartile range.

* *P* < 0.05

** *P* < 0.01.

## DISCUSSION

This quality improvement study explored how a clinical dietitian embedded to facilitate staff‐led enhancement of nutrition care at an orthopedic ward, using the Model for Improvement and small‐scale PDSA cycles, could enhance nutrition care for older patients with hip fractures.^15^ Although the study was limited by its modest scale and sample size, the observed improvements in nutrition risk screening, documentation of intake, and the proportion of patients meeting their individual energy and protein requirements indicate that structured, context‐adapted processes can drive meaningful change in complex clinical settings.

The intervention was codesigned and implemented in close collaboration with ward staff, ensuring feasibility and contextual relevance. This approach reflects key principles of pragmatic action research, in which improvements are shaped by those delivering care and adapted to real‐life conditions.^14^ Through two structured PDSA cycles, targeting nutrition documentation and energy and protein intake, the study demonstrated how iterative, staff‐led change can identify barriers, test solutions, and embed new practices into everyday routines.

Similar results were seen in a study by Mortensen et al, in which PDSA cycles improved adherence to vitamin D and calcium supplementation guidelines in nursing homes. The positive outcomes were achieved by addressing known barriers and actively engaging staff, which enabled the successful integration of new practices into existing workflows.^18^

These findings support previous research by Crowl et al, highlighting the Model for Improvement's value in fostering change through staff engagement and continuous measurement.^15^ Likewise, Taylor et al emphasize that the model's effectiveness depends on iterative testing, data‐driven decisions, and adaptation to the local context.^19^

Importantly, this study offers practical knowledge on how a low‐resource, staff‐driven model can address implementation gaps in nutrition care for a patient group at high risk of malnutrition. This is particularly critical because older adults with hip fractures frequently fail to meet basic nutrition requirements during hospitalization, thereby increasing their risk of adverse outcomes, delayed recovery, and elevated healthcare costs.^7^, ^20^, ^21^ In this context, even modest interventions that systematically improve care processes hold a significant relevance.

A core principle of the Model for Improvement is the establishment of clear roles and responsibilities to ensure accountability and integration of change into daily clinical practice.^15^, ^19^ In our study, the structured role of the embedded clinical dietitian was essential to strengthening nutrition care. As Nielsen et al emphasize, when responsibility for nutrition is diffuse, it risks being deprioritized or inconsistently delivered.^22^ Their findings highlight the value of embedding clinical dietitians directly into ward teams to enhance collaboration, ensure consistent follow‐up, and support the integration of nutrition care into standard treatment. Therefore, rather than acting as peripheral consultants, dietitians should be regarded as core members of the interdisciplinary team.^22^

Although the significant improvements observed in screening, documentation, and nutrition intake are encouraging, the small sample size limits the ability to draw firm conclusions about the intervention's overall effectiveness. Nevertheless, this study suggests that by placing the nutrition responsibility in the hands of a designated professional, such as a clinical dietitian, and supporting this with clear managerial backing and interdisciplinary collaboration, nutrition care can be elevated from an overlooked task into a coordinated and clearly structured component of hospital practice. These findings are in line with previous research, including an interdisciplinary collaboration care model by Bell et al, which included improved intake, reduced nutrition decline, and increased discharge to home.^23^ Thorsen et al found that increased access to clinical dietitians was associated with greater attention to nutrition among physicians and nurses,^13^ whereas Hoekstra et al also demonstrated that a multidisciplinary care model including a clinical dietitian improved intake, nutrition status, and quality of life 3 months after surgery.^24^

Our findings, in line with previous studies, suggest that interdisciplinary collaboration and clearly defined roles, with the Model for Improvement used as the guiding framework for the improvement process, are key to advancing nutrition care in complex clinical settings. However, future studies should explore this model across longer timeframes, larger populations, and in other settings where multidisciplinary engagement is central to care delivery.

## STRENGTHS AND LIMITATIONS

A key strength of this study is its pragmatic and context‐sensitive design, which allowed the intervention to be developed and tested under real‐life conditions. The use of action research and iterative PDSA cycles ensured continuous adaptation and strong engagement of frontline staff, supporting high relevance, feasibility, and ownership. Although the single‐site design limits generalizability, the structured approach may be transferable to other hospital settings with similar nutrition care challenges. The small sample size, pre‐post design, and absence of nutrition intake data at the immediate preintervention baseline limit the ability to attribute effects solely to the intervention. Nevertheless, the structured use of two clearly defined cycles, with adjustment based on real‐time observations, remains a methodological strength. The short intervention period further limits conclusions about long‐term sustainability, and the lack of patient and relative involvement constrains relevance. Future initiatives should integrate consumer perspectives and additional drivers such as mobilization before meals and antinausea medication, which may also affect intake. Finally, recruitment and data collection were conducted by the embedded dietitian, which may represent a potential source of bias. However, as she stepped back from her supportive role during data collection, the results primarily reflect the staff's independent practice while still capturing the effect of her educational role.

## CONCLUSION

This study suggests that using the Model for Improvement to support closer collaboration between a clinical dietitian and ward staff can enhance the interdisciplinary nutrition care processes. The intervention was associated with improved screening, documentation of nutrition intake, and increasing nutrition intake in patients with hip fractures at nutrition risk. These findings contribute to the growing evidence that structured, dietitian‐supported interventions, when adapted to the clinical context and integrated into daily practice, can strengthen hospital nutrition care.

## AUTHOR CONTRIBUTIONS

Tina Munk, Anne Marie Beck, Anne W. Knudsen, Cecilie M. Møller, Frederikke E. Pudselykke, Guro Ø.H. Mikkelsen, Heidrun T. Filtenborg, Trine S. Pedersen, and Jens Peter Alva‐Jørgensen contributed to conception or design of the study. Anne W. Knudsen, Cecilie M. Møller, Frederikke E. Pudselykke, Tina Munk, and Anne Marie Beck contributed to the acquisition, analysis, or interpretation of data. Tina Munk drafted the manuscript. Anne Marie Beck, Anne W. Knudsen, Cecilie M. Møller, Frederikke E. Pudselykke, Guro Ø.H. Mikkelsen, Heidrun T. Filtenborg, Trine S. Pedersen, and Jens Peter Alva‐Jørgensen critically revised the manuscript. All authors gave final approval and agreed to be accountable for all aspects of work, ensuring integrity and accuracy.

## CONFLICT OF INTEREST STATEMENT

None declared.

## Supporting information

Munk 2025.

## References

[1] Dempewolf S , Mouser B , Rupe M , Owen EC , Reider L , Willey MC . What are the barriers to incorporating nutrition interventions into care of older adults with femoral fragility fractures? Iowa Orthop J. 2023;43(2):172‐182. 38213858 PMC10777707

[2] Dagnelie PC , Willems PC , Jørgensen NR . Nutritional status as independent prognostic factor of outcome and mortality until five years after hip fracture: a comprehensive prospective study. Osteoporos Int. 2024;35(7):1273‐1287. 10.1007/s00198-024-07088-3 38760504 PMC11211177

[3] Wilkinson BR , An Q , Glass N , Miller A , Davison J , Willey MC . Malnutrition is common and increases the risk of adverse medical events in older adults with femoral fragility fractures. Iowa Orthop J. 2022;42(1):69‐74. 35821930 PMC9210413

[4] Malafarina V , Reginster JY , Cabrerizo S , et al. Nutritional status and nutritional treatment are related to outcomes and mortality in older adults with hip fracture. Nutrients. 2018;10(5):555. 10.3390/nu10050555 29710860 PMC5986435

[5] Hoekstra JC , Goosen JHM , de Wolf GS , Verheyen CCPM . Effectiveness of multidisciplinary nutritional care on nutritional intake, nutritional status and quality of life in patients with hip fractures: a controlled prospective cohort study. Clin Nutr. 2011;30(4):455‐461. 10.1016/j.clnu.2011.01.011 21342737

[6] Delmi M , Rapin CH , Bengoa JM , Delmas PD , Vasey H , Bonjour JP . Dietary supplementation in elderly patients with fractured neck of the femur. Lancet. 1990;335(8696):1013‐1016. 1970070 10.1016/0140-6736(90)91073-j

[7] Frederiksen AKS , Beck AM , Luiking YC , Hofstede JM , Knudsen AW , Munk T . Protein intake in hospitalized older patients after hip fracture: pilot feasibility study evaluating ESPEN guidelines for geriatrics. Clin Nutr Open Sc. 2022;42:148‐159. 10.1016/j.nutos.2022.03.001

[8] Avenell A , Handoll H . Nutritional supplementation for hip fracture aftercare in the elderly. Cochrane Database Syst Rev. 2004;11(11):CD001880. 10.1002/14651858.cd001880.pub2 14973973

[9] Volkert D , Beck AM , Cederholm T , et al. ESPEN guideline on clinical nutrition and hydration in geriatrics. Clin Nutr. 2019;38(1):10‐47. 10.1016/j.clnu.2018.05.024 30005900

[10] Bell J , Turabi R , Olsen SU , Sheehan KJ , Geirsdóttir ÓG . Interdisciplinary oral nutrition support and supplementation after hip fracture surgery in older adult inpatients: A Global Cross‐Sectional Survey (ONS‐STUDY). Nutrients. 2025;17(2):240. 10.3390/nu17020240 39861370 PMC11767526

[11] Beck AM , Balknäs UN , Camilo ME , et al. The European view of hospital undernutrition. Nutr Clin Pract. 2003;18(3):247‐249. 10.1177/0115426503018003247 16215044

[12] Duncan DG , Beck SJ , Hood K , Johansen A . Using dietetic assistants to improve the outcome of hip fracture: a randomised controlled trial of nutritional support in an acute trauma ward. Age Ageing. 2006;35(2):148‐153. 10.1093/ageing/afj011 16354710

[13] Thoresen L , Rothenberg E , Beck AM , Irtun Ø . Doctors and nurses on wards with greater access to. J Hum Nutr Diet. 2008;21(3):239‐247. 18477179 10.1111/j.1365-277X.2008.00869.x

[14] Bell JJ , Rossi T , Bauer JD , Capra S . Developing and evaluating interventions that are applicable and relevant to inpatients and those who care for them: a multiphase, pragmatic action research approach. BMC Med Res Methodol. 2014;14(1):98. 10.1186/1471-2288-14-98 25135226 PMC4150929

[15] Crowl A , Sharma A , Sorge L , Sorensen T . Accelerating quality improvement within your organization: applying the model for improvement. J Am Pharm Assoc (2003). 2015;55(4):e364‐e376. quiz e375 10.1331/JAPhA.2015.15533 26163594

[16] Danish Health Authority . *Malnutrition: detection, treatment*, *and follow‐up of citizens and patients at nutritional risk. guidance for municipalities, hospitals, and general*; 2022. www.sst.dk (In Danish).

[17] Munk T , Bruun N , Nielsen MA , Thomsen T . From evidence to clinical practice: positive effect of implementing a protein‐enriched hospital menu in conjunction with individualized dietary counseling. Nutr Clin Pract. 2017;32(3):420‐426. 10.1177/0884533616688432 28145792

[18] Mortensen C , Tetens I , Kristensen M , Beck AM . Vitamin D and calcium supplementation in nursing homes—a quality improvement study. Nutrients. 2022;14(24):5360. 10.3390/nu14245360 36558519 PMC9780874

[19] Taylor MJ , McNicholas C , Nicolay C , Darzi A , Bell D , Reed JE . Systematic review of the application of the plan‐do‐study‐act method to improve quality in healthcare. BMJ Qual Saf. 2014;23(4):290‐298. 10.1136/bmjqs-2013-001862 PMC3963536 24025320

[20] Bell J , Bauer J , Capra S , Pulle CR . Barriers to nutritional intake in patients with acute hip fracture: time to treat malnutrition as a disease and food as a medicine? Can J Physiol Pharmacol. 2013;91(6):489‐495. 10.1139/cjpp-2012-0301 23746263

[21] Skogli E , Halvorsen C . *The Socioeconomic Consequences of Malnutrition in Denmark*. Menon Economics; 2024. (In Danish)

[22] Nielsen LP , Thomsen KH , Alleslev C , Mikkelsen S , Holst M . Implementation of nutritional care in hospitals: a qualitative study of barriers and facilitators using implementation theory. Scand J Caring Sci. 2024;38(3):657‐668. 10.1111/scs.13255 38520146

[23] Bell JJ , Bauer JD , Capra S , Pulle RC . Multidisciplinary, multi‐modal nutritional care in acute hip fracture inpatients—results of a pragmatic intervention. Clin Nutr. 2014;33(6):1101‐1107. 10.1016/j.clnu.2013.12.003 24388594

[24] Hoekstra JC , Goosen JHM , de Wolf GS , Verheyen CCPM . Effectiveness of multidisciplinary nutritional care on nutritional intake, nutritional status and quality of life in patients with hip fractures: a controlled prospective cohort study. Clin Nutr. 2011;30(4):455‐461. 10.1016/j.clnu.2011.01.011 21342737

