# Supplementary material for: The effects of a dietitian‐supported multidisciplinary nutrition intervention on optimizing nutrition care in older patients with hip fracture and at nutrition risk—A quality improvement study
Source: Nutr Clin Pract. 2025 Oct 8;40(6):1529–37. doi: 10.1002/ncp.70049 (PMC12590340; doi:10.1002/ncp.70049)
Supplement: Supplementary file 1 — Munk 2025. [file NCP-40-1529-s001.pptx]

## Slide 1
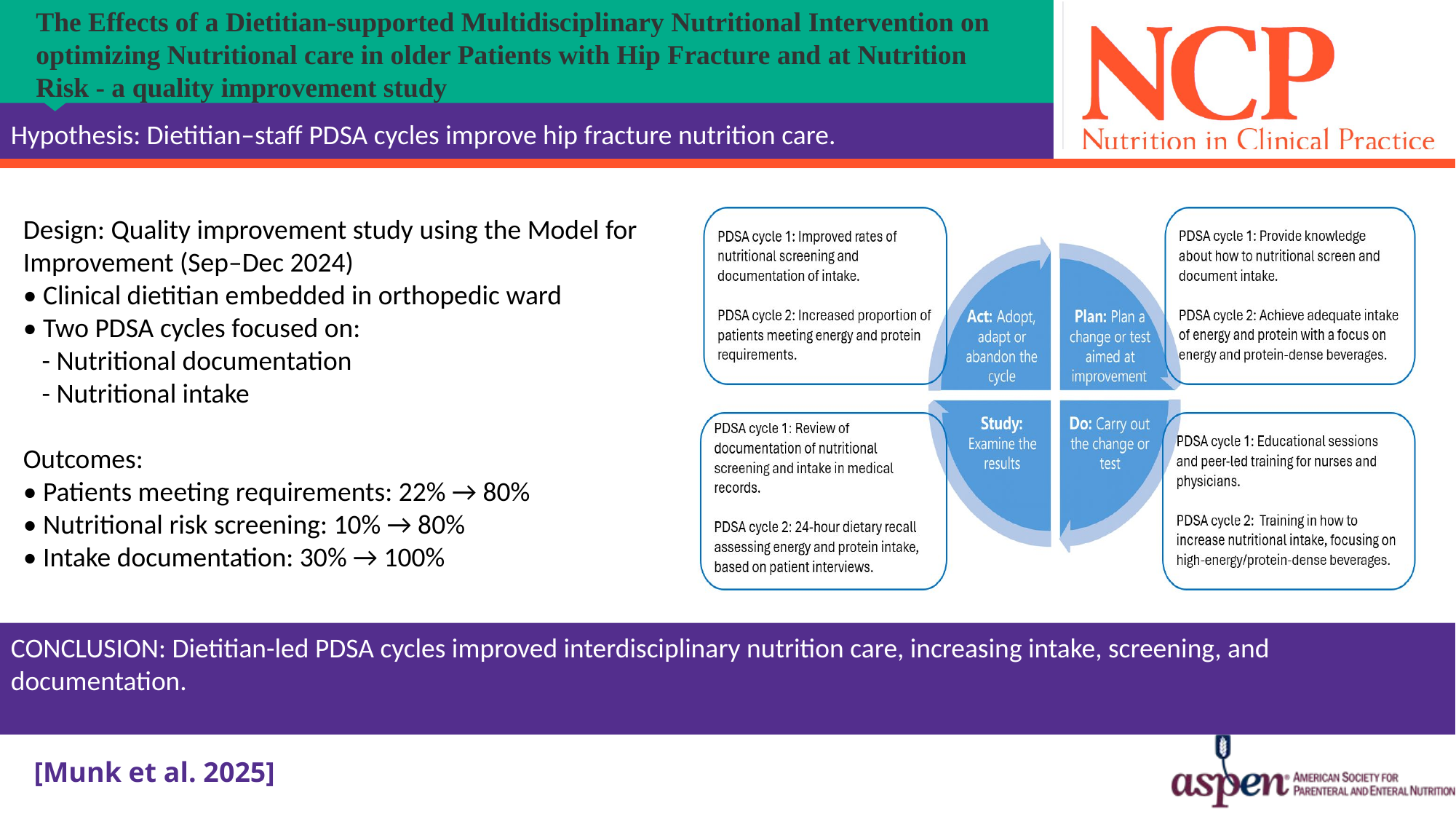

The Effects of a Dietitian-supported Multidisciplinary Nutritional Intervention on optimizing Nutritional care in older Patients with Hip Fracture and at Nutrition Risk - a quality improvement study
Hypothesis: Dietitian–staff PDSA cycles improve hip fracture nutrition care.
Design: Quality improvement study using the Model for Improvement (Sep–Dec 2024)
• Clinical dietitian embedded in orthopedic ward
• Two PDSA cycles focused on:
 - Nutritional documentation
 - Nutritional intake
Outcomes:
• Patients meeting requirements: 22% → 80%
• Nutritional risk screening: 10% → 80%
• Intake documentation: 30% → 100%
CONCLUSION: Dietitian-led PDSA cycles improved interdisciplinary nutrition care, increasing intake, screening, and documentation.
[Munk et al. 2025]
